# Laparoscopic roux-en-Y gastric bypass versus sleeve gastrectomy for teenagers with severe obesity - TEEN-BEST: study protocol of a multicenter randomized controlled trial

**DOI:** 10.1186/s12893-020-00778-9

**Published:** 2020-06-03

**Authors:** Daniëlle S. Bonouvrie, Andrew J. Beamish, Wouter K. G. Leclercq, Edgar G. A. H. van Mil, Arijan A. P. M. Luijten, Eric J. Hazebroek, Anita C. E. Vreugdenhil, Torsten Olbers, François M. H. van Dielen

**Affiliations:** 1 Obesity Center Máxima – Máxima Medical Center, Eindhoven/Veldhoven, The Netherlands; 2 Department of Gastrosurgical Research and Education – Institute of Clinical Sciences, Gothenburg University, Gothenburg, Sweden; 3 Department of Research, Royal College of Surgeons of England, London, United Kingdom; 4 Department of Pediatrics – Jeroen Bosch Hospital, ‘s-Hertogenbosch, The Netherlands; 5 Department of Surgery, Rijnstate Hospital, Arnhem, The Netherlands; 6 Department of Pediatrics, Maastricht University Medical Center, Maastricht, The Netherlands; 7 Department of Biomedical and Clinical Sciences and Wallenberg Center for Molecular Medicine, Linköping University, Linköping, Sweden; 8 Department of Surgery – Vrinnevi hospital, Norrköping, Sweden

**Keywords:** Adolescents, Bariatric surgery, Sleeve gastrectomy, Roux-en-Y gastric bypass, Severe obesity

## Abstract

**Background:**

Recent data support the use of bariatric surgery in adolescents with severe obesity following unsuccessful non-surgical treatments. Sleeve gastrectomy (SG) and Roux-en-Y gastric bypass (RYGB) have demonstrated reasonably similar weight loss and reduction of obesity related comorbidities in randomized trials in adults. SG has internationally become the most commonly used procedure in adolescents, yet long-term outcome data are lacking. No randomized controlled trial comparing SG and RYGB has been performed in adolescents.

**Objective:**

Determine whether SG is non-inferior to RYGB in terms of total body weight (TBW) loss in adolescents with severe obesity.

**Methods:**

A multicenter randomized controlled non-inferiority trial. Two hundred sixty-four adolescents aged 13–17 (Tanner stage ≥IV) with severe obesity (corrected for age and sex) will be included. Adolescents agreeing to participate will be randomized to either RYGB or SG. The primary outcome is the proportion of participants achieving 20% TBW loss at 3 years postoperatively. Secondary outcomes include (i) change in body weight, body mass index (BMI) and BMI standard deviation score, (ii) incidence of adverse health events and need for additional surgical intervention, (iii) resolution of obesity-related comorbidities, (iv) prevalence of cardio metabolic risk factor measures, (v) bone health measures and incidence of bone fractures, (vi) quality of life including psychosocial health, patient satisfaction and educational attainment and (vii) body composition. Follow-up will extend into the long term.

**Results:**

Not applicable.

**Discussion:**

This study will, to our knowledge, be the first randomized controlled trial comparing SG and RYGB in adolescents with severe obesity.

**Trial registration:**

The trial is registered at the Netherlands Trial Register on July 26th, 2018 – NTR7191 - https://www.trialregister.nl/trial/7191 (protocol version 5.0 – February 3th 2020).

## Background

The prevalence of overweight and obesity in adults is still increasing worldwide. Parallel to this, the prevalence of overweight and obesity in children has increased by almost 50% between 1980 and 2013 [1]. The majority of children with obesity will remain affected into their adult life [2], especially those with severe obesity post-pubertally [3].

Obesity is a chronic disease associated with several comorbidities including Type 2 diabetes (T2D), cardiovascular disease (including hypertension and dyslipidemia), musculoskeletal disorders and some cancers [4]. Correlated to the increase in prevalence of childhood obesity, an alarming shift in the onset of these obesity related comorbidities towards childhood has been noted, particularly for T2D [1, 5–10]. The timeframe between the onset of T2D and the requirement of insulin therapy is shorter in adolescents than in adults, with medical treatments failing earlier [7, 8]. Additionally, other comorbidities including metabolic disturbances also develop earlier in life [10]. All these factors contribute to a poor prognosis in adolescents with severe obesity, in whom studies have indicated a reduction in life expectancy of almost 20 years [5, 11, 12].

The standard treatment for obesity in children consists of multimodal lifestyle intervention programs, delivered by an expert multidisciplinary team focusing on eating patterns, exercise and behavior. An updated Cochrane Review meta-analyzed 37 studies, including a total of 27,946 children, concluded that there is strong evidence for the beneficial effects of multimodal lifestyle intervention programs for childhood obesity. Results included a mean reduction of 0.15 kg per meter squared (kg/m^2^) in body mass index (BMI). However, the reduction in the adolescent group (aged 13–18 years) was only 0.09 kg/m^2^ [13]. A study from the Netherlands showed that a multimodal lifestyle intervention program resulted in significant weight loss and improvement of cardiovascular risk parameters in children with overweight, obesity and severe obesity, all to a similar degree. In children with severe obesity a decrease in BMI z-score of − 0.23 ± 0.32 (*p* = 0.01) was observed after 2 years. Overall, 68% percent of the participants achieved a successful weight reduction, defined as 10% weight loss at 24 months follow-up [14]. However, despite these promising results, as much as one quarter do not experience weight reduction, which mainly applies to adolescents [14, 15]. Lifestyle intervention is thus not a solution for a subgroup of adolescents with severe obesity, whereas comorbidity is high in this group, urging for other intervention possibilities. Bariatric surgery should be studied as an option.

A recent systematic review of medium- and long-term outcomes (minimum three-year follow-up) of bariatric surgery including 950 adolescents with severe obesity, aged twelve to nineteen years, showed an average decrease in BMI of 13.3 kg/m^2^. Resolution of T2D/insulin resistance, hypertension and dyslipidemia occurred in 69.9, 61.6 and 57.1% of patients respectively. The rate of reoperation was 9.6%, mostly because of postoperative complications and suboptimal weight loss [16]. Olbers et al. reported similar weight loss results over 5 years among adolescents and adults who received a Roux-en-Y gastric bypass (RYGB), with a mean BMI-reduction of 13.1 kg/m^2^ in the adolescent intervention group. Notably, the control group of adolescents, who attended multimodal lifestyle intervention programs, experienced a mean increase in BMI of 3.3 kg/m^2^ across the five-year study period. Regarding comorbidities among adolescents who received the RYGB, resolution of hypertension was seen in 100%, resolution of dyslipidemia in 82.7% and complete resolution of T2D and disturbed glucose homeostasis in 100% (*n* = 3) and 85.7%, respectively [17]. In recent years, Inge et al. have published three-year outcomes from the Teenage Longitudinal Assessment of Bariatric Surgery (Teen-LABS) prospective longitudinal study including adolescents undergoing sleeve gastrectomy (SG) and RYGB. This study showed a mean three-year BMI reduction of 15 kg/m^2^ after RYGB and 13 kg/m^2^ after SG. Furthermore, significant improvements were observed in weight related quality of life and cardio-metabolic health (95% remission of T2D, 86% remission of abnormal kidney function, 74% remission of elevated blood pressure, 76% remission of prediabetes and 66% remission of dyslipidemia). This study suggested that risks associated with the procedures may be more prevalent after RYGB and included specific micronutrient deficiencies and the need for additional abdominal procedures [18]. The Teen-LABS group subsequently published 5-year outcomes after RYGB in comparison to adults in a similar study, LABS (Longitudinal Assessment of Bariatric Surgery). This confirmed similar weight loss outcomes between adolescents and adults, but a more favorable T2D and hypertension outcome in adolescents, supporting the case for early intervention [19].

The rates of remission of comorbidities after bariatric surgery observed in each of the previous mentioned studies were higher than those reported in adults, suggesting that adolescents may have a greater potential than adults for reversal of the cardio-metabolic consequences of obesity [17–19]. In addition, Panunzi et al. showed in the Swedish Obese Subjects study of adult patients that when T2D diagnosis was new (< 1 year) bariatric surgery resulted in > 90% remission, whereas a diagnosis of T2D > 4 years ago resulted in less than 40% remission [20]. Therefore, delay of surgical treatment until adulthood is negatively associated with the reduction of several comorbidities, cardiovascular risk profile and premature death.

In short, among adolescents with severe obesity who do not respond sufficiently to multimodal lifestyle interventions, bariatric surgery is a viable option. Although both SG and RYGB showed successful weight loss and reduction of obesity related comorbidities in adolescents thus far, long-term outcome data of SG in adolescents has been limited and, to date, no randomized controlled trial (RCT) has been performed in adolescents directly comparing these two procedures. This clear knowledge gap hampers optimal procedure selection for adolescents and thus prevents evidence-based recommendation to eligible adolescents. Therefore, we propose an RCT comparing SG with RYGB in adolescents with severe obesity.

## Methods/design

### Hypothesis

SG in combination with lifestyle intervention is non-inferior to RYGB in combination with lifestyle intervention in terms of proportion of participants achieving a total body weight (TBW) loss of at least 20% at 3 years, with an equivalent or lower rate of complications.

### Objective

The main objective of this trial is to obtain level one evidence regarding differences in clinical outcomes between RYGB and SG (both performed as add on to lifestyle intervention) in adolescents with severe obesity. By assessing efficacy and safety we aim to provide guidance regarding procedure choice based on reliable risk/benefit data overall as well as in subgroups.

### Trial design

**Fig. 1:**
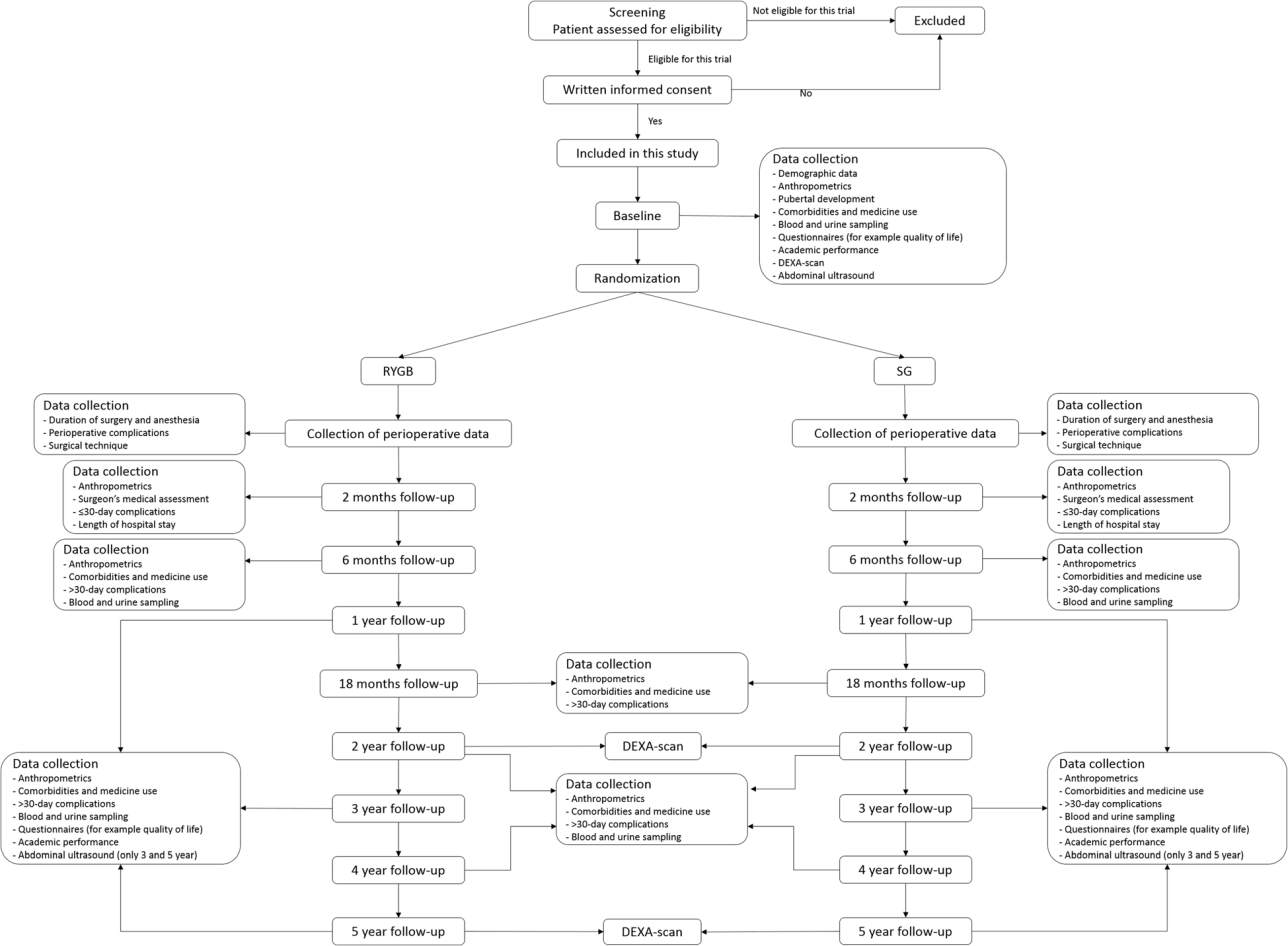
TEEN-BEST flowchart and participant timeline. RYGB = Roux-en-Y Gastric Bypass, SG = Sleeve Gastrectomy, DEXA-scan = Dual-energy X-ray Absorptiometry scan

This trial is designed as a non-inferiority, parallel, international multicenter, randomized controlled trial, comparing two bariatric surgeries (RYGB and SG) in adolescents with morbid obesity. The TEEN-BEST flow-chart, including the participant timeline, is shown in Fig. 1.

The trial consists of two phases. Phase 1 will be an internal pilot of twenty patients at each of the two initiating surgical sites (10 + 10 SG and 10 + 10 RYGB in total) to establish feasibility. The methods of recruitment and informed consent will be refined over this period. Phase 2 will be the full multicenter RCT. Patients will be recruited starting in May 2020 until May 2023. Follow-up within the RCT will be planned for a minimum of 5 years.

A matched group, identified from historical data in the multimodal lifestyle intervention program (COACH) of Maastricht University Medical Center, will be used as a non-surgical comparator to the bariatric procedures.

### Study setting

This study will initially be conducted at two surgical sites (a non-academic Dutch center and an academic Swedish center), with the potential to recruit additional sites after successful initiation. All surgical centers will collaborate with a child obesity center, which will initially be three sites (one academic and one non-academic Dutch center and one academic Swedish center). The list of study sites can be obtained from the corresponding author (bariatrics.resurge@mmc.nl).

### Informed consent procedure

Participants will be identified by collaborators in the child obesity lifestyle programs at the participating child obesity centers or by pediatricians outside of these centers. Existing patients who have already participated for 12 months in a child obesity lifestyle program or prospective identification of new patients (minimum of 12 months in a lifestyle program) with the potential to meet eligibility will be screened for recruitment.

During a multidisciplinary meeting eligible patients will be identified. The multidisciplinary team will, as a minimum, consist of a pediatrician, a clinical psychologist, a dietician and a bariatric surgeon. Patients identified as eligible for this study during the multidisciplinary meeting will be informed by their pediatrician about the trial. All patients will receive a written information leaflet and be required to provide written informed consent. When an adolescent is interested in participating in this trial, the pediatrician will ask the individual permission for the coordinating investigator to contact them providing more information about the trial and informed consent.

Informed consent will be obtained in the outpatient clinic prior to the participant undergoing any procedure that is specifically performed for the purposes of the trial at the participating site, including the collection of identifiable participant data. This informed consent conversation will be performed with the coordinating investigator. In adolescents, aged 13–14-15 years, we will additionally obtain informed consent from either both parents or caregivers. The informed consent form of this study is attached as Supplemental Appendix File 1.

### Study population

All willing patients who meet age, Tanner stage and BMI criteria, and have participated for at least 12 months in a lifestyle intervention program, will be offered formal assessment for study inclusion. Patients who continue to meet eligibility criteria will be invited to be included in the trial.

#### Inclusion criteria

(i) Completed a minimum of 12 months in multidisciplinary lifestyle intervention and/or pharmacotherapy weight loss program;
(ii) Consensus in the multidisciplinary child obesity team on the diagnosis of suboptimal outcome (defined as a TBW loss of < 10% after 12 months) following multidisciplinary lifestyle interventions;
(iii) Age 13–17 years;
(iv) Severe obesity meeting International Federation for the Surgery of Obesity and metabolic disorders (IFSO) criteria for bariatric surgery: BMI ≥40 kg/m^2^ with minor comorbidities, or BMI ≥35 kg/m^2^ with at least one major comorbidity, corrected for age and sex according to the International Obesity Task Force (IOTF) criteria.
(v) Tanner stage ≥IV;
(vi) Consensus in the multidisciplinary child obesity team, during the multidisciplinary meeting, on a motivated participation during the lifestyle intervention program so far, likely to persist in the future. The participant must demonstrate commitment to a bariatric program in the knowledge that they will be expected to continue with this effort after the bariatric surgery;

#### Exclusion criteria

(i) Unable to consent as appropriate;
(ii) Illiteracy (inability to read and understand questionnaires);
(iii) Secondary obesity – obesity caused by a uncontrolled medical condition;
(iv) Known syndrome or genetic disorder (such as Prader-Willi syndrome);
(v) Skeletal immaturity (Tanner stage ≤III) – pre-menarche – bone age < 15 years in boys;
(vi) Ongoing addiction (alcohol, drugs, medication);
(vii) Previous bariatric, gastro-esophageal reflux or gastric surgery;
(viii) Uncontrolled psychiatric disorder;
(ix) Inflammatory bowel disease;
(x) Non-support of both parents / caregivers.

### Eligibility criteria study centers and bariatric surgeons

Surgical sites are required to have (i) a bariatric team performing at least 300 adult bariatric procedures yearly, (ii) an existing child obesity management program, or a close link with such a program in another institute and (iii) an intensive care unit that treats adolescents, or access to such a facility nearby.

The bariatric surgeon(s) will be required to have a minimum experience of at least fifty of each bariatric procedure (SG and RYGB) in adults.

### Intervention

Eligible participants will be randomized to receive either RYGB or SG (both in combination with lifestyle intervention). To assure high quality of both bariatric procedures surgical protocols have been written and will be monitored during the trial.

All patients included in the study will have a protein diet (Modifast or Weight care) 2 weeks prior the surgery, with a standard number of calories per day (approximately 600 cal). This very low-calorie diet is given in order to decrease liver volume and increase laparoscopic workspace. It is proven that this very low-calorie diet reduces the postoperative complication rate in patients who underwent a laparoscopic RYGB [21].

Patients are required to take vitamins daily for the rest of their life (including extra Calcium+ Vitamin D, Vitamin B12 and Iron) according to local guidelines.

#### Laparoscopic RYGB

The surgical procedure has been described earlier by Dillemans et al. (circular anastomosis) [22] and by Lönroth et al. (linear anastomosis) [23]. In short, after induction of pneumoperitoneum and placement of five laparoscopic ports the majority of the stomach is disconnected from the normal digestive route using a linear stapler to leave a small (20–25 ml) gastric pouch in continuity with the esophagus. The jejunum is transected approximately 100 cm (cm) from the ligament of Treitz and the distal end (Roux limb) is anastomosed to the gastric pouch, as a gastrojejunal anastomosis, using a 25 mm circular stapler or a 30–45 mm linear stapler. Thereafter, the proximal end (the biliary limb) is attached approximately 50–150 cm distally along the jejunum, as a jejuno-jejunal anastomosis. Furthermore, the mesenteric defects beneath the jejunojejunostomy and at Petersen’s space will be closed. Before closure of the skin incisions, the gastrojejunostomy is tested for leakage using methylene blue and an easy flow drain (optional) will be placed. After closure of the incisions bupivacaine will be injected subcutaneously.

#### Laparoscopic SG

The procedure was performed by Gagner [24]. SG involves the excision of the majority of the stomach on its greater curvature side, using a stapling device. In short, pneumoperitoneum is induced with the Verres needle and five laparoscopic ports are placed, as with the laparoscopic RYGB. The resection line begins from approximately five centimeters proximal to the pylorus, proceeding to the angle of His to result in a tube or sleeve-shaped remnant stomach of approximately 25% its original capacity. A calibration bougie, usually sized between 34 and 40 Fr, is used to standardize the sleeve size. Before closure, the stomach remnant will first be removed and the gastric tube will be tested for leakage with methylene blue. Furthermore, after closure of the incisions bupivacaine will be injected subcutaneously. Placement of an easy flow drain is optional.

### Outcomes

#### Primary outcome measure (non-inferiority)

The proportion of patients that achieve a TBW loss of at least 20% at 3 years after surgery.

#### Secondary outcome measures

To compare outcomes between SG and RYGB. In addition, a historical cohort of adolescents who participated in a lifestyle intervention program only will be compared to both study arms on these secondary objectives (except for (ii) and (vii)).

(i) BMI, BMI standard deviation score and TBW loss [1, 3 and 5 years after the bariatric surgery]: weight loss is measured in kilogram and as percentage TBW loss;
(ii) Adverse health events [1, 3 and 5 years after the bariatric surgery]: including complications (within 30 days and beyond 30 days of bariatric procedure) and the need for re-operation. Complications will be scored according to the Clavien Dindo Classification [25];
(iii) Resolution of co-morbidities [1, 3 and 5 years after the bariatric surgery]: blood pressure (systolic and diastolic), lipid profile (HDL, LDL, TG), glucose control (HbA1c, fasting blood glucose level, fasting insulin, HOMA-IR), Obstructive Sleep Apnea (OSA) (Epworth sleepiness scale), kidney function (Glomerular Filtration Rate, microalbuminuria, creatinine), liver disease (i.e. non-alcoholic fatty liver disease) (alkaline phosphatase, gamma glutamyl transpeptidase, aspartate-aminotransferase, alanine-aminotransferase, bilirubin, ultrasound (baseline, 3 and 5 years post-surgery) and decrease/change in medication for each of the co-morbidities;
(iv) Resolution of OSA, T2D and pre-diabetes, hypertension, dyslipidemia, deranged liver function [1, 3 and 5 years after the bariatric surgery];
(v) Prevalence of cardio-metabolic risk factor measures [1, 3 and 5 years after the bariatric surgery];
(vi) Routine post-bariatric surgery nutritional blood tests at each assessment [1, 3 and 5 years after the bariatric surgery]: including full blood count, electrolytes, creatinine, fasting glucose, fasting insulin, HbA1c, liver parameters and function tests, iron, ferritin, vitamin B12, thiamine, folate/red cell folate, lipid profile, 25-hydroxyvitamin D, calcium and parathyroid hormone;
(vii) Bone health measures and the incidence of bone fractures [baseline and at 2 and 5 years after the bariatric surgery]: bone mineral density (DEXA-scan), osteocalcin, PINP, CTX and bone-specific alkaline phosphatase;
(viii) Generic and obesity-specific health-related quality of life [1, 3 and 5 years after the bariatric surgery]: IWQOL-Lite, RAND-36, Kidscreen-27;
(ix) Psychosocial health measures and educational attainment [1, 3 and 5 years after the bariatric surgery]: education, depression (Beck Depression Inventory), anxiety (Beck Anxiety Inventory), self-esteem (Kidscreen-27, IWQOL-Lite), attention deficit hyperactivity disorder (AVL);
(x) Patient satisfaction [1, 3 and 5 years after the bariatric surgery]: single question scale 1–10 and net promotor score;
(xi) Body composition [1, 3 and 5 years after the bariatric surgery]: DEXA-scan.

### Sample size calculation

A clinical successful weight loss is defined as a TBW loss of ≥20% for this study. We obtained unpublished summary statistics from the Teen-LABS study group, which were used to inform the power calculation. The proportion of participants losing at least 20% of their total body weight at 3 years in the Teen-LABS study was 63% after SG and 72% after RYGB.

The power calculation requires the estimation of two parameters, i.e. the mean TBW loss of participants at 3 years and the difference in mean TBW loss that would be considered clinically important (the non-inferiority margin). The hypothesis is that 70% of the participants will achieve a TBW loss of 20%. The non-inferiority margin was chosen on the basis of the opinions of the clinical applicants and was set at 20%.

A group sample size of 132 patients/arm, allowing for a 15% dropout, is needed to achieve 90% power to detect non-inferiority using a one-sided Z-test (unpooled). The non-inferiority margin is − 0.20000. The true difference between the means is assumed to be 0. The significance level (alpha) of the test is 0.02500.

### Randomization

Randomization will be performed by the coordinating investigator after trial eligibility and informed consent to participate in the trial have been confirmed. Randomization will be performed using a computerized randomization program (Research Manager), which will produce unchangeable computer-generated numbers. Randomization will be stratified according to centers in order to ensure balanced groups. Randomization will be on a 1:1 basis using block sizes of 6–8 participants.

### Blinding

Patients and caregivers (but not the surgical team) will be blinded to which procedure was performed until the two-month follow-up visit, which gives unbiased data regarding the 30-day complications. Standardized management, appropriate to both SG and RYGB, will be conducted during the blinded period and dietary advice and supplementation appropriate to both procedures will be administered to all patients.

Within the first 2 months, the trial code will only be broken in exceptional circumstances when knowledge of the surgical technique is essential for the safety of the patient. If unblinding is required, a formal request for unblinding must be made. The principle investigator (PI) will enter Research Manager for unblinding and will contact the holder of the code break list as a back-up. The coordinating investigator will notify the Sponsor in writing as soon as possible following the code break including the reason(s) for the code break.

### Data collection, data management and data analysis

After written informed consent is obtained, all patients will be assigned a study number. This moment is defined as baseline; the date when the participant is examined and found suitable to be randomized. Data will be pseudonymous inserted into a computerized database, Research Manager Software (certified by the ‘Information Security Management System 27001’), by local investigators. All data will be handled confidentially, anonymously and in accordance with the international accepted Personal Data Protection Act. A subject identification list will be drafted. This list will be password protected. The subject identification list and the password will be administered by the coordinating and principal investigator.

The follow-up of patients that withdrew from the study will continue according to the implemented standard care for adults who underwent bariatric surgery. This actually means that the follow-up just continues according to the follow-up schedule of the study, because the follow-up in the study is according to the standard care (including laboratory assays and quality of life assessment) for adults.

Primary analyses will be based on intention-to-treat and will include all randomized patients. In addition, a per-protocol analysis will be performed to explore the influence of protocol deviations and compare the results with the primary analysis. Furthermore, to explore the influence of contamination (switching between study arms) we will perform an as-treated analysis, the results of which will be compared with the results of the primary analysis.

The primary parameter, the proportion of patients achieving at least 20% TBW loss at 3 years, will be compared using descriptive statistics and a logistic regression analysis.

Secondary parameters, including quality of life questionnaire scores and other continuous outcomes measured at multiple time points, will be compared using a mixed regression model with baseline and post-surgery measures modeled jointly. Changes in treatment effect with time will be assessed by adding a treatment by time interaction to the model and comparing models using a likelihood ratio test. Time to event outcomes will be compared using survival methods for interval censored data. Frequencies of adverse events will be described. Treatment differences will be reported with 95% confidence intervals (CIs). A detailed analysis plan will be prepared during the feasibility phase 1.

We will compare outcomes between groups at the end of phase 1. Other interim analyses will be decided in discussion with the data safety monitoring committee (DSMC).

In addition, one subgroup analysis is planned; outcomes will be described for male and female participants. Differences in treatment effect between the two subgroups will be tested by including interaction terms to the analysis model.

Missing data will be excluded and will not be imputed. To address possible bias of the missing values, the baseline characteristics of patients with and without missing values will be compared. We will do our utmost to collect outcome measures wherever possible to minimize the number of missing values. This means we will also accept patient reported weight in case of missing weight data. In addition, we will try to retrieve the reason for the missing value, such as missing because of weight gain.

### Monitoring

The DSMC will review the data periodically regarding the safety and efficacy of the trial procedures and advise the sponsor on the future management of the trial. They will review any unexpected adverse event and may ask to review outcomes or other data that may have an impact on the trial.

They will perform interim analyses, can decide to end the study prematurely and will send their advice to the sponsor of the study. Should the sponsor decide not to fully implement the advice of the DSMC, the sponsor will send the advice to the reviewing REC, including a note to substantiate why the advice of the DSMC (or part thereof) will not be followed.

Independence is a key characteristic of this committee, where the committee members are completely uninvolved in the running of the trial and the committee members cannot be unfairly influenced by people or institutions involved in the trial. The members of the DSMC will reflect the disciplines necessary to interpret the data from the trial; an epidemiologist/statistician, a surgeon, a pediatrician and a bariatric surgeon with experience in adolescents.

All adverse events (AE), related to the bariatric surgery, reported spontaneously by the subject or observed by the investigator or his staff and are unexpected, and serious adverse events (SAE) will be recorded and reported to the sponsor by the main coordinating investigator in accordance with the International Conference for Harmonization of Good Clinical Practice guidelines and the Sponsor’s Research Related Adverse Event Reporting Policy. Abnormalities in blood- and urine samples will only be noted as an AE in case intervention is required. SAEs that are critical to the safety evaluation of the participant need to be reported directly (within 24 h) to the main coordinating investigator. The sponsor will report the AEs/SAEs to the Research Ethics Committee (REC) within 7 days (death or life-threatening) or within fifteen days.

The Clinical Trial Center Maastricht will perform the external monitoring audit of this study. The monitoring will be done in the first year and at the end of the study in all participating centers. In between, monitoring will be conducted using a risk-based approach that focuses on sites that have, for example, the highest enrolment rates, largest numbers of withdrawals, and/or the highest numbers of reported AEs or SAEs. Specific attention will be payed to SAEs, informed consent, data monitoring and completeness of case record forms.

### Ethics and dissemination

#### Research ethics approval

This study will be performed in accordance with the ethical standards of the institutional and/or national research committee (Medical Research Involving Human Subjects Act) and with the 1964 Helsinki declaration and its later amendments or comparable ethical standards. Informed consent will be obtained from all individual participants included in the study. The medical ethical reviewing committee Máxima Medical Center approved the TEEN-BEST study protocol and all participating centers on June 5th, 2018 and approved TEEN-BEST study protocol version 5.0 on April 1th, 2020 (REC number W18.015).

#### Protocol amendments

The accredited REC will be informed of all substantial amendments. They will be responsible for approval of the amendment prior to implementation in the protocol. After approval, the protocol amendments will be communicated with the local investigators and the Netherlands Trial Register.

#### Confidentially

The participant will be assigned a study number after randomization and a subject identification list will be drafted. This list will be password protected and will be administered by the coordinating investigator and PI. All data will be handled confidentially, anonymously and in accordance with the internationally accepted Personal Data Protection Act. Data will be inserted into a computerized database, Research Manager Software, by local investigators. Registration will be monitored and is in line with Good Clinical Practice guidelines.

Archiving of the trial documentation will be authorized by the sponsor following submission of the end of trial report. Data and samples from this study will be stored for a period of fifteen years after completion of the trial. Destruction of essential documents will require authorization from the sponsor.

#### Access of data

Access to the data will be limited to the research team (local investigators, coordinating investigator and PI), Inspection for Healthcare/audits, monitors and auditors in line with participant consent.

#### Declaration of interests

The authors declare that they have no competing interests.

#### Ancillary and post-trial care

Both the sponsors/investigators have a liability insurance, which is in accordance with article 7 of the WMO. The sponsors also have insurance in accordance with the legal requirements in the Netherlands (Article 7 WMO). This insurance provides cover for harm to research subjects through injury or death caused by the study. The insurance applies to harm that becomes apparent during the study or within 4 years after the end of the study.

#### Dissemination policy

Research data can only be presented and/or published with agreement from the PI. The research data will be reported following the Consolidated Standards of Reporting Trials guidelines.

## Discussion

Recent data support the use of bariatric surgery in adolescents with severe obesity as an additional treatment to lifestyle intervention. Although both SG and RYGB have demonstrated successful weight loss and reduction of obesity related comorbidities thus far, long-term outcome data of SG in adolescents have been scarce. No RCT has been performed in adolescents directly comparing these two bariatric procedures. This knowledge gap hampers optimal procedure selection in adolescents and prevents evidence-based recommendation to eligible adolescents.

TEEN-BEST will be the first randomized controlled trial comparing SG and RYGB integrated in the stepped/matched care of adolescents with severe obesity, thus combining the benefits of both multimodal lifestyle intervention and surgical intervention, and will guide future adolescent bariatric practice.

## Supplementary information

**Additional file 1.**

## Data Availability

The datasets used and/or analyzed during the current study are available from the corresponding author on reasonable request.

## Abbreviations

AE: Adverse Event
BMI: Body Mass Index
cm: Centimeter
DEXA: Dual-energy X-ray Absorptiometry
DSMC: Data Safety and Monitoring Committee
IFSO: International Federation for the Surgery of Obesity and Metabolic disorders
IOTF: International Obesity Task Force
Kg/m^2^: Kilogram per square meter
OSA: Obstructive Sleep Apnea
PI: Principle Investigator
RCT: Randomized Control Trial
REC: Research Ethics Committee
RYGB: Roux-en-Y Gastric Bypass
SAE: Serious Adverse Event
SG: Sleeve Gastrectomy
T2D: Type 2 Diabetes
TBW: Total Body Weight
