# Supplementary material for: Laparoscopic roux-en-Y gastric bypass versus sleeve gastrectomy for teenagers with severe obesity - TEEN-BEST: study protocol of a multicenter randomized controlled trial
Source: BMC Surg. 2020 Jun 3;20:117. doi: 10.1186/s12893-020-00778-9 (PMC7268239; doi:10.1186/s12893-020-00778-9)
Supplement: Supplementary file 1 — Additional file 1. [file 12893_2020_778_MOESM1_ESM.pdf]

Appendix File 1 – Informed consent form

TEEN-BEST

Gastric Bypass versus Gastric Sleeve bij jongeren (13 t/m 17 jaar) met ernstig overgewicht

Bijlage B – Toestemmingsformulier minderjarige proefpersonen van 12 t/m 17 jaar

Ik heb de informatiebrief gelezen. Ook kon ik aanvullende vragen stellen. Mijn vragen zijn voldoende beantwoord. Ik had genoeg tijd om te beslissen of ik meedoe.

Ik weet dat meedoen vrijwillig is. Ook weet ik dat ik op ieder moment kan beslissen om toch niet mee te doen of te stoppen met het onderzoek. Daarvoor hoef ik geen reden te geven.

Ik geef toestemming om mijn huisarts te vertellen dat ik meedoe aan dit onderzoek.

Ik geef toestemming voor het informeren van mijn huisarts en/of behandelend kinderarts over onverwachte bevindingen die van belang (kunnen) zijn voor mijn gezondheid.

Ik weet dat voor de controle van het onderzoek sommige mensen mijn gegevens kunnen inzien. Deze mensen staan vermeld in de informatiebrief. Ik geef toestemming voor die inzage door deze personen.

Ik geef toestemming voor het verzamelen en gebruiken van mijn gegevens op de manier en voor de doelen die in de informatiebrief staan.

Ik geef □ **wel** toestemming om mijn persoonsgegevens langer te bewaren en te gebruiken voor toekomstig onderzoek op het gebied van morbide obesitas.
Ik geef □ **geen** toestemming om mijn persoonsgegevens langer te bewaren en te gebruiken voor toekomstig onderzoek op het gebied van morbide obesitas.

Ik geef □ **wel** toestemming om in de toekomst (na afloop van dit onderzoek) benaderd te worden voor eventueel vervolgonderzoek.
Ik geef □ **geen** toestemming om in de toekomst (na afloop van dit onderzoek) benaderd te worden voor eventueel vervolgonderzoek.

Ik wil meedoen aan dit onderzoek.

Naam proefpersoon*:

Handtekening: Datum: __ / __ / __

Ik verklaar dat ik deze proefpersoon volledig heb geïnformeerd over het genoemde onderzoek.

Als er tijdens het onderzoek informatie bekend wordt die de toestemming van de proefpersoon zou kunnen beïnvloeden, dan breng ik hem/haar daarvan tijdig op de hoogte.

Naam onderzoeker (of diens vertegenwoordiger):

Handtekening: Datum: __ / __ / __

**Voor kinderen van 12 t/m 15 jaar, die zelfstandig beslissingen kunnen nemen (wilsbekwaam zijn), moeten de ouders/voogd ook Bijlage C ondertekenen.*

De proefpersoon krijgt een volledige informatiebrief mee, samen met een kopie van het getekende toestemmingsformulier.

TEEN-BEST

Gastric Bypass versus Gastric Sleeve bij jongeren (13 t/m 17 jaar) met ernstig overgewicht

Bijlage C – Toestemmingsformulier ouders of voogden

Ik ben gevraagd om toestemming te geven voor deelname van de volgende persoon/mijn kind aan dit medisch-wetenschappelijke onderzoek:

Naam proefpersoon (kind): Geboortedatum: __ / __ / __

Ik heb de informatiebrief voor ouders/voogden gelezen. Ook kon ik aanvullende vragen stellen. Mijn vragen zijn voldoende beantwoord. Ik had genoeg tijd om te beslissen of ik wil dat mijn kind meedoet.

Ik weet dat meedoen vrijwillig is. Ook weet ik dat ik op ieder moment kan beslissen dat mijn kind toch niet meedoet. Daarvoor hoef ik geen reden te geven.

Ik geef toestemming om de huisarts van mijn kind te informeren dat mijn kind meedoet aan dit onderzoek.

Ik geef toestemming voor het informeren van de huisarts en/of behandelend specialist van mijn kind over onverwachte bevindingen die van belang (kunnen) zijn voor de gezondheid van mijn kind.

Ik weet dat voor de controle van het onderzoek sommige mensen de gegevens van mijn kind kunnen inzien. Deze mensen staan vermeld in de informatiebrief. Ik geef toestemming voor die inzage door deze personen.

Ik geef toestemming voor gebruik van de gegevens op de manier en voor de doelen die in de informatiebrief staan (zie ook sectie 2 onder keuring).

Ik geef □ **wel** toestemming om de persoonsgegevens van mijn kind langer te bewaren en te gebruiken voor toekomstig onderzoek op het gebied van morbide obesitas.
Ik geef □ **geen** toestemming om de persoonsgegevens van mijn kind langer te bewaren en te gebruiken voor toekomstig onderzoek op het gebied van morbide obesitas.

Ik geef □ **wel** toestemming om mijn kind na dit onderzoek opnieuw te benaderen voor een vervolg–onderzoek.
Ik geef □ **geen** toestemming om mijn kind na dit onderzoek opnieuw te benaderen voor een vervolg–onderzoek.

Ik ga ermee akkoord dat deze persoon/mijn kind meedoet aan dit onderzoek.

Naam ouder/voogd*:

Handtekening: Datum: __ / __ / __

Naam ouder/voogd*:

Handtekening: Datum: __ / __ / __

Ik verklaar hierbij dat ik bovengenoemde persoon/personen volledig heb geïnformeerd over het genoemde onderzoek.

Als er tijdens het onderzoek informatie bekend wordt die de toestemming van de ouder of voogd zou kunnen beïnvloeden, dan breng ik hem/haar daarvan tijdig op de hoogte.

Naam onderzoeker (of diens vertegenwoordiger):

Handtekening: Datum: __ / __ / __

----

* Als het kind jonger dan 16 jaar is, ondertekenen de ouders die het gezag uitoefenen of de voogd dit formulier. Kinderen van 12 t/m 15 jaar die zelfstandig beslissingen kunnen nemen (wilsbekwaam zijn), moeten daarnaast zelf een formulier ondertekenen, zie bijlage B.

De ouder/voogd krijgt een volledige informatiebrief mee, samen met een kopie van de getekende versie van het toestemmingsformulier.
